# Is early goal-directed therapy associated with a higher risk of adverse events?

**DOI:** 10.1186/s40560-018-0345-1

**Published:** 2018-11-26

**Authors:** Ahmad Sabry Saleh

**Affiliations:** Intensive Care Unit, Okba Ben Nafee Hospital, 45 street, el-Asafra, Alexandria, 21539 Egypt

**Keywords:** Septic shock, Early-goal directed therapy, Usual care, Serious adverse events

## Abstract

The Japanese Clinical Practice Guidelines for Management of Sepsis and Septic Shock 2016 suggested against the use of the early goal-directed therapy (EGDT) in patients with septic shock. This recommendation was based on the three large-scale trials (ProCESS, ARISE, and ProMISe). Although the three trials showed no difference in mortality between EGDT and usual care, the guidelines determined that the potential harms presented by EGDT likely outweigh its potential benefits. On the contrary, analysis of data from the three trials showed an approaching statistical significance lower risk of serious adverse events in the EGDT group compared to usual care (risk difference = − 1%, 95% confidence interval; − 2% to 0%, *P* = 0.05). EGDT may still be beneficial in patients with high disease severity and low central venous oxygen saturation, especially when managed by less experienced staff.

Dear Editor,

I read with great interest the English edition of the Japanese Clinical Practice Guidelines for Management of Sepsis and Septic Shock 2016 published recently in the *Journal of Intensive Care* [1]. Although the guidelines are primarily tailored to the Japanese context, it represents an excellent summary of the current literature and thus it is of great interest to intensivists from around the globe. I would like to discuss a few points regarding section CQ7: Initial resuscitation/inotropes.

First, the guidelines suggested against the use of the early goal-directed therapy (EGDT) when performing initial resuscitation in patients with sepsis or septic shock. EGDT is a 6-h resuscitation protocol for the administration of intravenous fluids, vasopressors, inotropes, and red-cell transfusion to achieve pre-specified targets for central venous pressure, arterial blood pressure, urine output, and central venous oxygen saturation (ScvO_2_) [2]. This recommendation was based on the three large-scale randomized controlled trials (RCTs); ProCESS [3], ARISE [4], and ProMISe [5] reported in 2014 and 2015. Although the three RCTs showed no significant difference in mortality between EGDT and usual care (i.e., equipoise), the guidelines determined that the potential harms presented by EGDT likely outweigh its potential benefits and explained their rationale as follows: “Dobutamine dosages and the quantity of blood transfused increased significantly in the EGDT group, and due to the increased frequency of arrhythmias associated with dobutamine, greater overall risk of side effects associated with transfusions, and increased time and quantity of work required of hospital staff, it is possible that compliance with EGDT may increase the risk of harm (burden) faced by patients” [1].

The notion that EGDT may increase the risk of harm to patients is rather speculative and not supported by clinical evidence or patients’ data. On the contrary, pooled data from the three RCTs (Fig. 1) showed an approaching statistical significance lower risk of serious adverse events (SAEs) in the EGDT group compared to usual care (risk difference = − 1%, 95% confidence interval; − 2% to 0%, *P* = 0.05). SAEs were uniformly defined among the three RCTs as “any untoward medical occurrence that: (1) results in death, (2) is life-threatening, (3) requires in-patient hospitalization or prolongation of existing hospitalization, (4) results in persistent or significant disability/incapacity, (5) is a congenital anomaly/birth defect, or (6) other adverse event considered serious by medical judgment” [3–5].

**Fig. 1:**
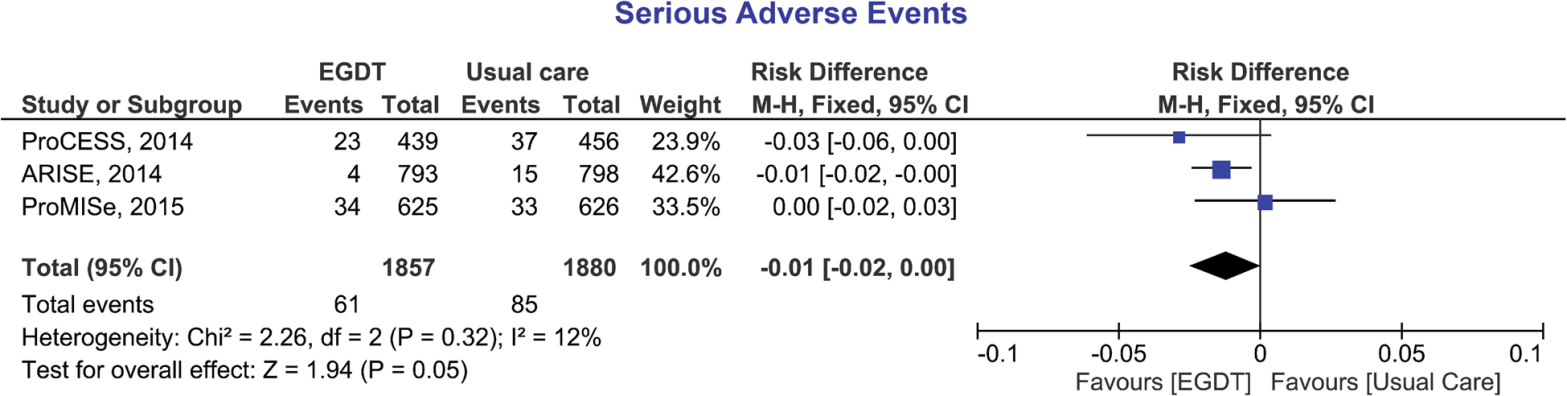
Forest plot of the risk difference of serious adverse events (SAEs) between early goal-directed therapy (EGDT) and usual care. The risk difference of individual studies is represented by a *square* through which runs a *horizontal line* (95% confidence interval). The *diamond* represents the pooled effect size. Data were extracted from the supplementary appendix of each trial and analyzed by RevMan 5. Events refer to number of SAEs not the number of patients (some patients might experience more than one SAE). *ProCESS* reported all SAEs occurring in the first 72 h post-randomization and after 72 h SAEs were limited to a specific list of events that could be related to the intervention at that point in time (e.g., central line infection, or arterial line complication), or events that the site principal investigator considered potentially related to the study intervention. *ARISE* reported SAEs up to 72 h post-randomization only. *ProMIse* reported SAEs within 30 days

Second, the guidelines provided some contradictory statements. Despite the recommendation against EGDT, the guidelines provided an expert consensus statement that either ScvO_2_ or lactate clearance may be used as indicators of initial resuscitation (CQ7–8). And suggested that Dobutamine is used in septic shock when cardiac function remains diminished, and maintenance of hemodynamics is insufficient despite adequate fluid resuscitation and noradrenaline administration (CQ7–12) [1]. Both ScvO_2_ monitoring and Dobutamine are cardinal components of the EGDT. ScvO_2_ monitoring (with the subsequent use of Dobutamine and red-cell transfusion to correct ScvO_2_) was the only intervention not allowed by study protocol in the usual care group in the three RCTs [3–5]. Thus, the only conclusion we could draw from the three RCTs is that catheter placement for continuous ScvO_2_ monitoring is not necessary in every patient presented by septic shock. However, the three RCTs could not answer the question of whether targeting ScvO_2_ of ≥ 70% is an effective intervention or not as the most of the patient were at target ScvO_2_ on presentation (initial mean ScvO_2_ was 71%, 72%, and 70% in the ProCESS [3], ARISE [4], and ProMISe [5], respectively). Until future trials focusing on the subgroup of patients with low ScvO_2_ is conducted, the evidence from the original EGDT trial [2] which recruited patients with low ScvO_2_ (mean 49%) is enough to consider the use of Dobutamine and red cell transfusion to correct ScvO_2_ to decrease mortality.

Finally, I totally agree with the guidelines statement that “the treatment of sepsis can vary significantly depending on the level of care offered by a given facility and the level of knowledge and skills of the attending physician and staff”. The Three RCTs [3–5] were primarily conducted in academic/tertiary care centers in high-income countries and included patients with low severity septic shock who rapidly responded to therapy. Even though, their usual care was associated with a trend toward higher risk of SAEs. The question here is, what would be the situation in less equipped facilities or with physicians with less expertise? In fact, multiple issues have been raised regarding the external validity of the three trials suggesting that EGDT may still be beneficial in patients with high disease severity and low ScvO_2_, especially when managed by less experienced staff who may appreciate using simple protocols [6, 7].

## Abbreviations

EGDT: Early goal-directed therapy
RCT: Randomized controlled trial
SAEs: Serious adverse events
ScvO_2_: Central venous oxygen saturation
