# Supplementary material for: Is early goal-directed therapy associated with a higher risk of adverse events?
Source: J Intensive Care. 2018 Nov 26;6:78. doi: 10.1186/s40560-018-0345-1 (PMC6260769; doi:10.1186/s40560-018-0345-1)
Supplement: Related texts

# Reply: Response to Dr. Saleh: “Is early goal-directed therapy associated with a higher risk of adverse events?”

**Authors:** Yasuyuki Kakihana, Asako Matsushima, Osamu Nishida

**Affiliations:** 2 Department of Emergency and Intensive Care Medicine-Kagoshima, University Graduate School of Medical and Dental Sciences, Kagoshima, Japan; 3 0000 0001 0943 978X grid.27476.30 Department of Advancing Acute Medicine, Nagoya University Graduate School of Medical Sciences, Nagoya, Japan; 4 0000 0004 1761 798X grid.256115.4 Department of Anesthesiology and Critical Care Medicine, Fujita Health University School of Medicine-Toyoake, Aichi, Japan

We read the letter to the editor from Dr. Saleh with great interest. For the assessment of the CQ7–1 “Is early goal-directed therapy (EGDT) recommended for initial resuscitation in patients with sepsis or septic shock?”, three randomized controlled trials (RCTs) [3–5] were identified based on a search of the PubMed database and were used in the final analysis. Our analyses showed no significant difference in mortality between EGDT and the standard treatment (90-day mortality rate: risk ratio 0.98 [95% confidence interval {CI} 0.88–1.10]; 28-day mortality rate: risk ratio 0.98 [95% CI 0.84–1.13]) [1]. Regarding the benefit-risk balance, dobutamine dosages and the quantity of blood transfused were significantly increased in the EGDT group compared to the usual-care group [3, 4], and due to the increased frequency of arrhythmias associated with dobutamine and greater overall risk of side effects associated with transfusions, it is possible that compliance with EGDT may increase the risk of harm (burden) faced by patients. As Dr. Saleh suggested first, however, the pooled data from the three RCTs show an approaching statistical significance lower risk of serious adverse events (SAEs) in the EGDT group compared to the usual-care group (risk difference = − 1%, 95% CI − 2–0%, *P* = 0.05). We therefore examined the data on the SAEs and AEs of the three RCTs again in detail and obtained the following results: In the ProCESS trials, reports of potential SAEs (excluding death) were rare and did not differ significantly across groups, with 23 events (5.2%) reported in the EGDT group and 37 (8.1%) in the usual-care group [3]; in the ProMISe trials, there was no significant between-group difference in the number of SAEs, with 34 events (5.4%) reported in the EGDT group and 33 (5.3%) in the usual-care group and at least 1 SAE reported among 30 patients (4.8%) in the EGDT group and 26 patients (4.2%) in the usual-care group (*P* = 0.58) [5]; a significant difference in the number of SAEs was observed only in the ARISE trials, with 4 events (0.5%) reported in the EGDT group and 15 (1.9%) in the usual-care group, although there was no significant between-group difference in the number of patients with ≥ 1 AE (including SAEs) (56 patients [7.1%] in the EGDT group and 42 patients [5.3%] in the usual-care group; *P* = 0.15) [4]. Special precautions should be taken when interpreting the findings for SAEs in the ARISE trials, as (1) the number of SAEs in the ARISE trials was too small for a comparison with those of the other RCTs (ProCESS and ProMISe), and (2) the reason for the large difference between the number of SAEs and the number of AEs (including SAEs) in the ARISE trial was unclear. SAEs may develop less frequently in the EGDT group than in the usual-care group, as suggested by Dr. Saleh.

Second, Dr. Saleh claimed that until future trials focusing on the subgroup of patients with a low ScvO_2_ are conducted, the evidence from the original EGDT trial [2] that recruited patients with a low ScvO_2_ (mean 49%) was sufficient to support the consideration of the use of dobutamine and red-cell transfusion to correct the ScvO_2_ in order to reduce mortality [7]. Because the serum lactate levels and ScvO_2_ are important for both assessing the oxygen transport capacity in tissues and as a marker of tissue hypoperfusion [8, 9], it is suggested that either factor (ScvO_2_ or lactate clearance) may be used as an indicator of initial resuscitation [10]. Recently, a meta-analysis of individual patient data from the three recent trials (ProCESS, ARISE, ProMISe) was designed prospectively to improve the statistical power and explore the heterogeneity of the treatment effect of EGDT. The results showed that there was no evidence of a benefit associated with EGDT in the subgroups with the most severe septic shock, including those with a serum lactate level of ≥ 4.1 mmol/L (mean, 6.7 mmol/L), those who presented with both hypotension and hyperlactatemia (mean systolic blood pressure, 89 mmHg; mean serum lactate level, 6.7 mmol/L), those in the upper third of APACHE II scores (mean score, 24.6), and those in the upper third of predicted risk of death [11]. We therefore cannot conclusively say that EGDT is beneficial for the most severe septic shock patients with hyperlactatemia (ScvO_2_ may be low).

Even if EGDT was associated with a lower incidence of SAEs than the usual-care and could be managed by less experienced staff who might appreciate using simple protocols, the recommendations for CQ7–1 in our guidelines remain unchanged. One of the most important principles to understand in the management of these complex patients is the need for a detailed initial assessment and ongoing reevaluation of the response to treatment. The use of CVP alone to guide fluid resuscitation, which is required by the EGDT protocol proposed by Rivers et al. [2], can no longer be justified [12] because the ability to predict a response to a fluid challenge when the CVP is within a relatively normal range (8–12 mmHg) is limited [13], and some patients treated with EGDT develop subsequent fluid overload, which may be associated with worse outcomes [14]. Dynamic measures of assessing whether or not a patient requires additional fluid have been proposed in an effort to improve fluid management and have demonstrated better diagnostic accuracy at predicting those patients who are likely to respond to a fluid challenge by increasing stroke volume. Furthermore, in recent years, the “hour-1 bundle” has been recommended, in which obtaining blood for measuring lactate and blood cultures, administering fluids and antibiotics, and in the case of life-threatening hypotension, initiating vasopressor therapy should all be begun immediately [15].

In their review, Nguyen et al. [6] stated that in this era of global reductions in sepsis mortality, clinicians should view EGDT as a verb (series of actions) rather than a noun. Thus, taking over the basic concept of EGDT to promptly improve the hypoperfusion of tissues but in keeping with the change in the new era, achieving the target value within 1 h (not 6 h) may require developing a new version of EGDT (modified EGDT) that not only selects dynamic indices instead of CVP to predict fluid responsiveness but also avoids the fluid overload following initial resuscitation.
